# Heart rate response to cannabis in active older adults

**DOI:** 10.1007/s00421-026-06152-6

**Published:** 2026-02-12

**Authors:** Ellie Sundali, Jonathon K Lisano, Angela D. Bryan, Carillon J. Skrzynski

**Affiliations:** 1 Department of Psychology and Neuroscience, University of Colorado Boulder, 1777 Exposition Dr, Boulder, CO 80301 USA; 2 Institute of Cognitive Science, University of Colorado Boulder, 1777 Exposition Dr, Boulder, CO 80301 USA

**Keywords:** Heart rate, Cannabis, Aging, Physical activity, Cardiovascular disease

## Abstract

**Purpose:**

Cannabis use is increasing among older adults. Given reported associations between delta-9-tetrahydrocannabinol (THC) and increased acute heart rate, there is concern regarding the impact of cannabis on cardiovascular health in older adults. Engaging in regular physical activity (PA) is associated with reduced heart rate and cardiovascular disease risk, but it has yet to be explored whether PA status influences the acute effects of THC on heart rate in older adults.

**Methods:**

This data is from a larger observational study of older adults using cannabis for sleep, pain, or mood problems. Based on participants’ self-report Leisure-Time Activity Categorical (L-Cat) responses, they were grouped into high, moderate, or low PA groups. Participant heart rate was measured pre- and 2 h post edible cannabis consumption. Self-reported THC dose (mg) was recorded, and blood THC was assessed pre- and 2 h post-consumption.

**Results:**

Two separate ANOVA models, controlling for age, assessed interactions between L-Cat group and blood or self-reported THC on change in heart rate pre- to post-use. The L-Cat group x self-reported THC interaction was not significant, but the L-Cat group x blood THC interaction was (F(2,202) = 6.45, partial *η*2 = 0.06, *p* = 0.002) such that higher blood THC was associated with a greater increase in heart rate, an effect most pronounced in the low PA group.

**Conclusion:**

These results align with previous research where THC is associated with higher heart rate but provide novel insight as to the potential protective effects of high levels of PA in older adults.

**Clinical trial registration:**

The study was pre-registered on Clinicaltrials.gov (NCT05188404).

## Introduction

Recreational cannabis is quickly becoming more available across the United States. It is currently available for purchase in 24 states, 3 territories, and the District of Columbia (National Conference of State Legislatures (NCSL) 2023). This spread in legalization has led to increased rates of use across most demographics and age groups (Farrelly et al. 2023), with older adults appearing to be the quickest-growing demographic of all (Kaskie et al. 2017; Han and Palamar 2020).

Heart disease, a subset of cardiovascular disease, is the leading cause of death in the United States in people 65 and older (Centers for Disease Control and Prevention [CDC] (2024)). Given the prevalence of how rapidly cannabis use is growing in the older adult demographic (Kaskie et al. 2017; Han and Palamar 2020), it is imperative to explore whether cannabis use alters this group’s cardiovascular disease risk. While determining cardiovascular disease risk consists of a multitude of factors, resting heart rate is easily measured and gives some indication of general cardiovascular health. At rest, a heart rate greater than 80 beats per minute (bpm) is linked to increased cardiovascular disease risk, while a heart rate less than 60 bpm is associated with lower cardiovascular disease-linked mortality (Saxena et al. 2013).

Previous research demonstrates that delta-9-tetrahydrocannabinol (THC), the primary active component in cannabis, dose-dependently increases heart rate in younger populations (Pabon et al. 2021; Muheriwa-Matemba et al. 2024). Physiologically, THC has been observed to reduce vagal tone and promote vasodilation (Latif and Garg, 2020), and both mechanisms are associated with acute increases in heart rate. Systematic review findings further indicate that tachycardia, a heart rate greater than 100 bpm, is the most common adverse cardiovascular effect in all routes of cannabis administration (i.e. smoking, oral ingestion, dabbing) and across all age groups including older adults (Muheriwa-Matemba et al. 2024). Unfortunately, the majority of existing empirical studies primarily focus on younger, healthier populations, emphasizing the importance of studying the effects of acute cannabis use specifically in older adults who are at greater risk for cardiovascular disease.

One factor that is known to reduce cardiovascular disease risk as well as resting heart rate is engagement in regular physical activity (PA) (Saxena et al. 2013). Unfortunately, only 27% of older adults meet the weekly exercise guidelines outlined by the American College of Sports Medicine (ACSM) (DiPietro 2019) including a minimum of 150 min of moderate-intensity physical activity or 75 min of vigorous-intensity activity per week. Physical inactivity is associated with more time spent watching TV, playing video games, or working at a computer (NCD Alliance 2025). A natural assumption is that cannabis use would similarly be associated with physical inactivity based on stereotypes surrounding cannabis use as being “demotivating” and causing “couch lock.” Interestingly, the data do not support these stereotypes. In fact, a number of studies suggest that people who use cannabis are actually more likely to be active than those who do not (e.g., Vidot et al. 2017; YorkWilliams et al. 2019) while others show no difference (Gibson et al. 2023). Further, a study from 2019 reported that 92% of participants who use cannabis in conjunction with PA had used cannabis within 1-hour of initiating exercise (Lisano et al. 2019). Given these findings, and the likelihood that there is some variability in PA among older adults who use cannabis, an interesting question is whether there is a possibility that increased levels of fitness indicated by greater PA might buffer the heart rate-increasing effects of cannabis use among this population who is vulnerable to cardiovascular disease.

The present study thus sought to explore changes in cardiovascular parameters (i.e. heart rate at rest) after acute cannabis use in older adults and to understand whether current PA status moderates these effects. To do this, we examined the relationship between heart rate, PA status, and THC amount (both self-reported dose (mg) and plasma THC concentrations (pg/mL)) during acute cannabis edible product use in 234 adults 60 years of age or older. Specifically, we hypothesized that the change in heart rate from immediately before use to 2 h after acute cannabis use would be greater as THC amount increased, and that this would differ based on PA status. However, we did not have specific hypotheses regarding the direction of the moderating effect of PA on this association due to a lack of existing evidence regarding the role of PA status in heart rate response to acute cannabis use.

## Methods

### Participants

Participants were recruited as part of a larger, NIH-funded longitudinal study examining cannabis use for sleep, pain, or mood problems among older adults (1R01AG066698; PI: Bryan) and was pre-registered on Clinicaltrials.gov (NCT05188404). The study was approved by the University of Colorado Boulder Institutional Review Board. Participants were recruited using mailed fliers, community advertisements, and social media. Interested participants were screened via an eligibility survey that was completed independently online or with a research assistant over the phone. Eligible participants were 60 years or older, able to provide informed consent, did not have a history/family history of psychosis or vertigo, were postmenopausal (if female), and did not engage in hazardous substance use (i.e., scores < 8 on the Alcohol Use Disorder Identification Task and screened negative for illicit drug use outside of cannabis at a baseline session). Participants also needed to be interested in using cannabis for pain, sleep problems, depression, or anxiety, but not be regular cannabis users. Regular cannabis use was defined as using more than seven times per month in the past 6 months. Participants were also excluded if they were already using cannabis to relieve symptoms more than once per month in the past 6 months. Older adults who had pain, sleep problems, depression, or anxiety but did not wish to use cannabis were included as a non-equivalent control group in the larger study, but their data are not included in the present analysis.

## Procedure

**Fig. 1:**
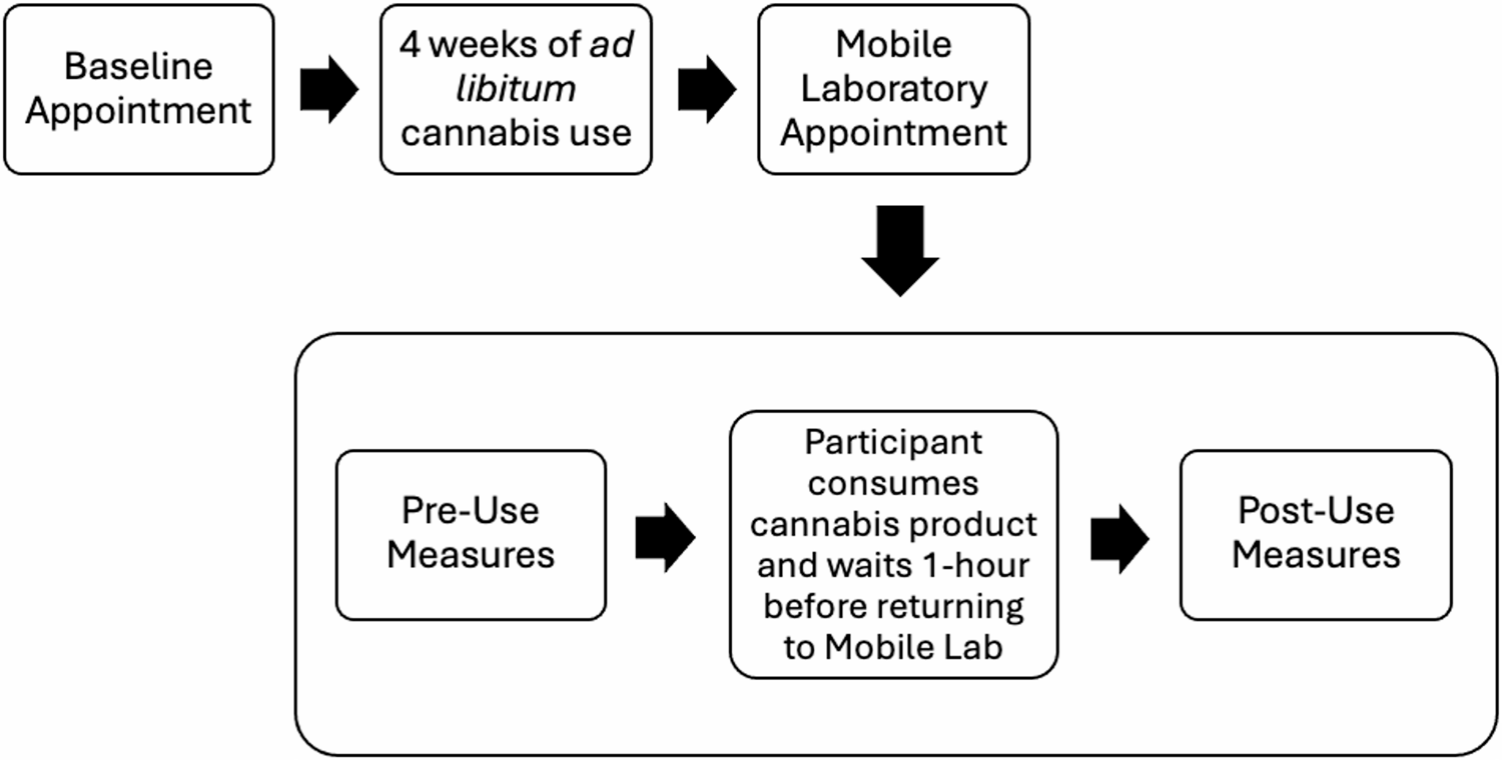
Study Visit Timeline. Note. Participants returned to the mobile laboratory after 1 h, but blood was collected 2 h post-consumption

Participants were asked to come to two in-person appointments over the course of 4 weeks, but only the data from the second appointment were used in the current analysis with the exception of PA status and demographic information (see below). Before each appointment, participants were asked to refrain from alcohol and cannabis use for 24 h. After completing an information session with a research assistant at the first appointment, participants were asked to go to a dispensary and chose a cannabis edible product to use *ad libitum* for 4 weeks. The participant then sent a picture of the cannabis product they purchased to an unblinded research assistant who would record the amount of THC and/or CBD in their product (mgs) into RedCap, a secure data storage system (Harris et al. 2009, 2019). After 4 weeks, research staff met participants at or near their home with our mobile pharmacology laboratory to assess measures before (pre-use) and after (post-use) the participant consumed as much of their chosen edible cannabis product as they wished within their home (see Fig. 1 for study timeline). Products contained varying amounts of THC. We utilized a mobile laboratory to comply with federal restrictions that prohibit the use of legal market cannabis products on university property due to schedule 1 classification of cannabis at the federal level (Hutchison et al. 2019). 

### Measures

#### Demographics

At baseline, participants completed information on age, sex, educational status, and race.

#### Physical activity (PA) status

Participants were grouped into different physical activity categories based on their response to the Leisure-Time Activity Categorical item (L-Cat; Kiernan et.al. 2013). This measure was completed at the baseline session as well as at the mobile laboratory session. Baseline values were utilized for categorization, but in cases where the data were missing from the baseline, the mobile lab data were used instead. The L-Cat consists of 1 item which assesses the average level of physical activity over the last month using a 1 to 6 scale. A score of “1” corresponded to the answer: “I did not do much physical activity. I mostly did things like watching television, reading, playing cards, or playing computer games. Only occasionally, no more than once or twice a month, did I do anything more active such as going for a walk or playing tennis,” with successive responses representing progressively higher levels of PA ending in “6” that corresponded to the answer: “Almost daily, that is five or more times a week, I did vigorous activities such as running or riding hard on a bike for 30 minutes or more each time.” Participants were included in the high physical activity group if they choose 5 or 6, moderate physical activity group if they choose 3 or 4, or low physical activity group if they choose 1 or 2.

#### THC amount

Ingested THC amount was self-reported by participants on the day of their mobile laboratory appointment (hereafter referred to as ingested THC). Specifically, participants were asked how much of their chosen product they planned on taking in terms of pieces (e.g., 1 gummy, ½ gummy, 3 drops, etc.) which the research experimenter recorded. Based on the product information gathered prior to the appointment, this information was used to calculate ingested THC.

Venous blood samples were collected by a certified phlebotomist at pre-use and 2 h post-use to quantify blood THC. Blood was collected 2 h post-use instead of 1 h, as previous studies indicate that cannabinoid levels peak around 1 h after oral administration, remain comparable at 2 h, then decline steadily over 2–3 h, dropping off after 4 h (Nadulski et al. 2005; Ohlsson et al. 1986). Thus, the 2-hour mark was chosen to account for metabolic differences and reduce participant discomfort. Blood was kept on ice until the mobile pharmacology laboratory returned to our laboratory on campus. Samples were then spun at 1000 rpm for 10 min, and separated plasma was aliquoted and stored at −80 °C until it was analyzed by the iC42 Laboratory at the University of Colorado Anschutz Medical Campus.

#### Heart rate

Heart rate in beats per minute (bpm) was measured using a Fabrication Enterprises, Inc. Beijing Choice Electronic Technology Co., Ltd. Baseline Pulse Oximeter (model 12–1926). Heart rate was measured after the participant had been sitting in a relaxed state, for at least 20 min while completing various cognitive tasks that required little movement. Heart rate was measured both before and two hours after edible product use and a difference score was used in analyses (i.e., heart rate pre-use – heart rate 2 h post-use).

## Data analysis

A priori power analyses were originally conducted for the larger study and indicated that a sample size of 287 participants would ensure 95% power to detect effects as small as f^2^ = 0.046, and 80% power to detect even smaller effects of f^2^ = 0.028. All analyses were conducted using R (R Core Team 2025). Preliminary analyses were first conducted to ensure PA groups did not differ on any relevant variables via analyses of variance (ANOVAs) and χ2 tests comparing demographics (e.g., age), cannabis use (e.g., days of use), and heart rate across timepoints. Primary analyses to examine if changes in heart rate after acute cannabis use differed based on PA status and THC level (i.e., two way interaction between THC x PA status) were conducted via analysis of variance (ANOVA) tests with PA group, ingested THC or 2 hour post-use blood THC levels (done in separate models to avoid issues of multicollinearity), and their interactions entered as predictors with change in heart rate as the outcome. Because age was significantly different across groups (see Results), it was included as a covariate. When a significant interaction was found, tests of simple slopes were conducted via the emmeans package (Lenth 2024) using the emtrends function and graphs were made via the ggplot2 package (Wickham et al. 2016) to aid in interpretation.

## Results

### Descriptives

Demographic data, cannabis use, and heart rate information for the three groups can be seen in Table 1.. Across the whole sample, participants were on average 70 years old (SD = 6.03), generally evenly split between males and females (54.27% female), mostly White (89.32%), well-educated, with the majority having at least a college degree (84.48%), and physically active, with the majority having a score of 3 or greater on the L-Cat On average, participants used cannabis 1 day a week at baseline. There were no differences between groups on any variable except for a small difference in age (F(2,231) = 3.43, *p* = 0.03), and, as would be expected given the heart rate lowering effects of PA, baseline heart rate (F(2,230) = 4.90, *p* = 0.01). Post hoc analyses indicated that the high PA group was younger than the moderate PA group, and baseline heart rate was lower among the high PA relative to the low PA group.

**Table 1:** Descriptive information across PA groups

	High PA (N=49)	Moderate PA (N=142)	Low PA (N=43)
Gender (%)			
Female	46.94	56.34	55.81
Male	53.06	43.66	44.19
Race (%)			
White	91.84	91.55	90.69
Asian	4.08	2.11	4.65
Black or African American	0.00	1.40	0.00
American Indian or Alaska Native	0.00	2.11	0.00
More than one race	0.00	1.40	0.00
Prefer not to answer	4.08	1.40	4.65
Education Status (%)			
Advanced degree	67.35	47.89	55.81
College degree	20.41	35.92	23.26
Some college or less	10.20	15.49	20.93
Age (M (SD))	68.00 (5.05)	70.56 (6.37)	69.47 (5.54)
Baseline heart rate (bpm) (M (SD))	63.35 (8.49)	67.48 (9.97)	69.81 (12.65)
Cannabis Use (M (SD))			
Ingested THC (mg)	4.33 (5.77)	3.4 (3.8)	4.19 (3.33)
Cannabis use days	1.14 (4.48)	0.99 (3.63)	0.86 (2.70)
Pre-use blood THC (pg/mL)	0.00 (0.00)	0.01 (0.15)	0.04 (0.22)
Post-use blood THC (pg/mL)	1.94 (3.14)	1.40 (1.89)	1.71 (2.30)

Note. THC mgs ingested were reported by the participant before using their product at the mobile laboratory visit. Pre- and post-use blood levels were collected at the van visit. Cannabis use days includes the 30 days prior to the baseline session

### Correlations

To explore the validity of self-reported THC amount during the mobile laboratory appointment, we looked at the correlation between ingested THC and THC assayed in blood. These values were significantly, positively correlated as expected (*r* = 0.62, *p* < 0.001).

### Primary analyses

#### Ingested THC

The ingested THC model resulted in a two-way interaction between THC amount and PA group that was just below the conventional threshold for significance (see Table 2). Post hoc tests indicated that with greater ingested THC, there was a larger increase in heart rate from pre- to 2 h post-use but only among individuals in the moderate PA group (trend = 0.54, standard error [SE] = 0.18, 95%CI = 0.19–0.89, *p* = 0.003), while this relationship was just below the conventional threshold for significance among those in the low PA group (trend = 0.65, SE = 0.39, 95%CI=−0.11−1.42, *p* = 0.09) and was non-significant in the high PA group (trend=−0.01, SE = 0.20, 95%CI=−0.41−0.38, *p* = 0.94).

**Table 2:** ANOVA findings predicting heart rate from THC, PA group, and their interactions

Predictors of heart rate	Ingested THC			Blood THC		
	F	Part η^2^	*p*- value	F	Part η^2^	*p*- value
PA Group	0.48	0.00	0.62	0.96	0.00	0.38
THC	2.84	0.03	0.09	**6.76**	**0.09**	**0.01**
Age	0.00	0.00	1.00	0.06	0.00	0.80
THC x PA Group	**2.50**	**0.02**	**0.08**	**6.45**	**0.06**	**0.002**

Values in bold are significant

#### Blood THC

**Fig. 2:**
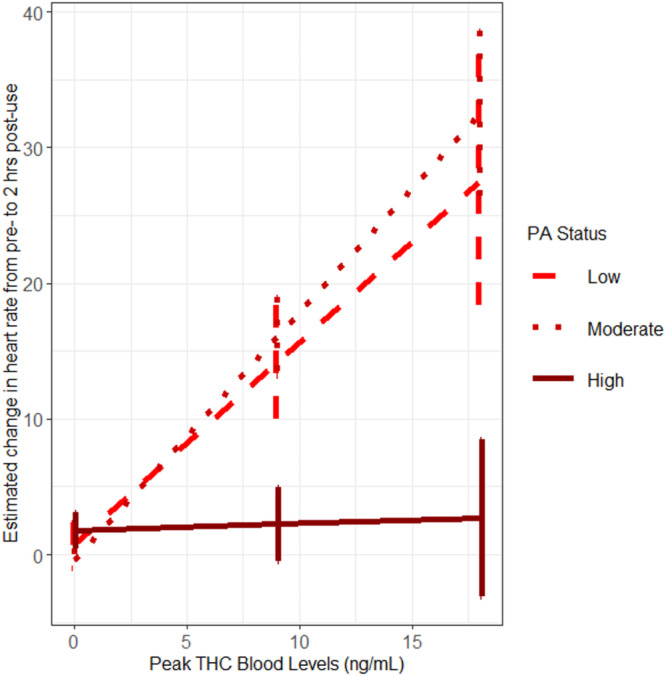
Relationship between blood THC and change in heart rate across PA groups

The pattern of findings was identical using blood THC, except here the THC X PA group interaction was significant (see Table 2). Post hoc analyses indicated that, increased THC blood levels were associated with greater increases in heart rate from pre- to 2 h post-use for individuals in the low PA group (trend = 1.49, SE = 0.57, 95%CI = 0.36–2.61, *p* = 0.01) and the moderate PA groups (trend = 1.82, SE = 0.37, 95%CI = 1.10–2.55, *p* < 0.001) but not the high PA group (trend = 0.05, SE = 0.35, 95%CI=−0.65−0.75, *p* = 0.89) (see Fig. 2).

## Discussion

This study is the first to report on the relationship between PA status and THC on changes in heart rate at rest following acute cannabis edible use among older adults. Analyses with both self-reported milligrams of ingested THC and with objective THC exposure assayed from blood resulted in an interaction between THC exposure and PA group. This effect showed that greater increases in THC were associated with greater increases in heart rate after acute edible cannabis use. However, this relationship was only observed in participants who were low or moderately active. Highly active participants experienced no significant changes in heart rate pre- to post-use, suggesting that higher levels of PA may have protective effects regarding acute elevations in heart rate associated with THC. These findings highlight that PA status seems to influence the acute heart rate response to THC among older adults.

It is intriguing that despite a high degree of correlation between ingested THC and blood THC that the two models assessed produced slightly different results. This discrepancy could in part be attributed to differences in absorption patterns and bioavailability of cannabis edible products. Previous research has reported that consuming a high-fat meal prior to cannabis edible consumption improves absorption via the gut and leads to higher blood THC concentration (Zgair et al. 2016). Although participants were asked to refrain from caffeine after consuming their edible cannabis product, they were not given specific instructions regarding what to eat while consuming their selected product in their home. The difference in the two models of this study indicate that the blood concentration of THC may be more reliable when assessing acute physiological effects of cannabis, especially when edible products are being used. Still, the present study findings on blood THC and associations with heart rate align with previous research in which acute THC use was related to higher heart rate in younger populations (Pabon et al. 2021; Muheriwa-Matemba et al. 2024) but add nuance in that high levels of PA may buffer this effect.

The present findings showed that highly active participants had little to no changes in heart rate regardless of changes in blood THC concentrations. It is well established that engaging in regular exercise is beneficial to cardiovascular health and is associated with lower resting heart rate (Reimers et al. 2018) which we also saw in this sample. The benefits of regular exercise also extend to the acute cardiovascular response to stress (i.e. exercise or cannabis use). For example, previous research has observed that physically fit individuals have lower heart rates when exercising at the same intensity as unfit individuals (Silverman and Deuster 2014; Webb et al. 2013; Sothmann et al. 1991). This is likely the result of more efficient cardiac function (Nystoriak and Bhatnagar 2018) and a dampened sympathetic response in physically fit individuals. Additionally, research supports that cannabis related tachycardia is due to sympathetic nervous system activation (Latif et al. 2020). It is plausible that the high PA group did not experience acute elevations in heart rate in response to THC due to better sympathetic regulation in response to physiological stress.

The acute naturalistic use of cannabis products available and widely used in the legal market in Colorado as well as the objective assessment of THC levels in blood are strengths of this study, but there are limitations that should be noted. Causal conclusions regarding the effects of different levels of THC on heart rate cannot be made because participants were allowed to self-select the type and amount of edible cannabis product they consumed. Thus, expectancy effects were not controlled for in this study and may be an alternative explanation for our findings. Additionally, the participants were largely white, affluent, and physically active, with 82% of participants considered moderately physically active based on scores of 3 or greater on the L-Cat, which limits generalizability. Future studies should consider incorporating an objective measure of PA to assess fitness in addition to the self-report measure, collect data on the timing of and types of food consumed that day, and utilizing a more diverse sample, both demographically and in terms of fitness levels. Additionally, future studies should prioritize randomized controlled trials that manipulate THC dose and product type to establish more concrete dose-response effects. Longitudinal studies should also follow individuals across repeated use sessions to examine the tolerance and adaptation effects that single acute use sessions cannot determine.

This study provided evidence that older adults who engage in low or moderate amounts of PA may be more susceptible to acute elevations in heart rate associated with the use of THC, while those with high levels of PA experience a buffering effect. While resting heart rate is only one aspect of cardiovascular health, it is a critical component. Thus finding ways to mitigate increases in heart rate after THC consumption, in this case via higher levels of PA, is important in the context of increasing cannabis use among older adults. Engagement in regular PA, which is already linked to numerous health benefits including promoting longevity, may also play an important role in reducing acute cardiovascular strain in response to THC.

## Data Availability

(1) The larger study which data was drawn from was pre-registered at Clinicaltrials.gov (NCT05188404), (2) analyses for the current paper were not formally pre-registered, (3-5) data, analytic code, and materials are available upon request to the corresponding author.
